# PROTS-RF: A Robust Model for Predicting Mutation-Induced Protein Stability Changes

**DOI:** 10.1371/journal.pone.0047247

**Published:** 2012-10-15

**Authors:** Yunqi Li, Jianwen Fang

**Affiliations:** Applied Bioinformatics Laboratory, The University of Kansas, Lawrence, Kansas, United States of America; Indian Institute of Science, India

## Abstract

The ability to improve protein thermostability via protein engineering is of great scientific interest and also has significant practical value. In this report we present PROTS-RF, a robust model based on the Random Forest algorithm capable of predicting thermostability changes induced by not only single-, but also double- or multiple-point mutations. The model is built using 41 features including evolutionary information, secondary structure, solvent accessibility and a set of fragment-based features. It achieves accuracies of 0.799,0.782, 0.787, and areas under receiver operating characteristic (ROC) curves of 0.873, 0.868 and 0.862 for single-, double- and multiple- point mutation datasets, respectively. Contrary to previous suggestions, our results clearly demonstrate that a robust predictive model trained for predicting single point mutation induced thermostability changes can be capable of predicting double and multiple point mutations. It also shows high levels of robustness in the tests using hypothetical reverse mutations. We demonstrate that testing datasets created based on physical principles can be highly useful for testing the robustness of predictive models.

## Introduction

The ability to improve protein thermostability via protein engineering is of great scientific interest and has significant practical value because many native proteins are only marginally stable under normal physiological and storage conditions [1]–[8]. For example, protein-based pharmaceuticals are often vulnerable to degradation that may affect their potency and even safety [9]. In addition, stable proteins are highly desirable in many biotechnological applications including biopharmaceuticals, biomaterials, and biofuel, etc. [7], [8]. Enzymes with enhanced stability allow catalyzed reactions to be performed at higher temperatures, which often lead to more efficient industrial processes.

Computational methods for designing proteins with enhanced thermostability can be advantageous over conventional approaches because of their potential low cost and time-saving properties [10]. Existing computational approaches use either force-fields [2], [11]–[16] or data mining technologies [17]–[25]. The former require high- resolution 3D structures and are often highly computer-intensive. Consequently, in recent years, data mining technologies employing various machine learning algorithms have increasingly attracted attention. The general procedure of machine learning approaches is to train predictive models based on available experimental data using features (properties) such as substitution types, secondary structures, solvent accessibility, and the amino acid composition of neighboring residues. Many algorithms including support vector machines [17]–[20], neuronal networks [21], and multiple regression and classification techniques [22], [23], have been used for predicting protein stability changes induced by mutations. The machine learning approaches hold great promises because they may be used to discover subtle patterns governing mutation induced stability changes and protein stability in general. However, recently we discovered that some of these types of methods may suffer from the over-fitting problem when hypothetical reverse mutations were used to test the robustness of these methods [26].

Usually protein stability changes upon mutations are experimentally measured through changes in the melting temperature (Δ*Tm*) or alterations of folding free energies (ΔΔ*G*) between wild type proteins and their mutants. Existing protein stability predictors use one or the other as the metric for stability changes. Because both metrics are thermodynamic parameters and thus also state functions [27], the ΔΔ*G* (or Δ*Tm*) of a mutation from a wild type protein to its mutant (WT−>MT) equals the negated ΔΔ*G*(or Δ*Tm*)of a hypothetical reverse mutation (MT−>WT), i.e.,
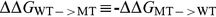
(1)

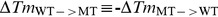
(2)


Our tests revealed that the tested methods lost predictive ability considerably when hypothetical reverse mutations were used to evaluate the robustness of these methods [26]. Our findings are consistent to the comprehensive analysis conducted by Khan and Vihinen recently. They evaluated and compared 11 online stability predictors and found that “at best, the predictions were only moderately accurate (∼60%)” [28]. Thus, effective and robust computational algorithms for predicting mutation induced protein stability change are still in critical demand.

In addition, most existing algorithms were developed for predicting thermostability changes of single-point mutations, despite the fact that the ability to predict protein stability changes upon multiple point mutations is also important because stabilization induced by single mutation may not be sufficient for practical applications of a protein. Only in recent years a few studies have been focused on multiple-mutation induced thermostability changes. For example, Huang and Gromiha proposed a predictive model named WET, a weighted decision table method for predicting protein thermostability change upon double mutation from amino acid sequences [23]. The model was built and tested on a set of 180 double point mutations. The correlation coefficient of the predicted and experimental ΔΔ*G* reached 0.75 and the overall accuracy was 82.2% in the 10-fold cross validation test [23]. However, the accuracy drops to 0.57 when it is tested on the hypothetical reverse mutations (see details in the results).

In this work, we attempt to develop a robust algorithm that can treat free energy as a thermodynamic parameter for predicting not only single-, but also multiple- point mutation induced thermostability change. A prerequisite for such a model is a set of suitable features relevant to the protein stability. We use several types of features for this study. The first type of features is the evolutionary information extracted from the target proteins since the “survival of the fittest” principle may be also applicable to protein thermostability. In fact, a concept of evolutionary pseudo free energy upon mutations was introduced and was found to have statistically significant correlations with protein thermostability changes [29]. Other features include secondary structures and solvent accessibility, either assigned based on structures or predicted by PSIPRED [30], depending on the availability of structures. In addition, we include features that we previously developed in ThermoRank [31], and a set of fragment-based thermostability terms [26].

In the following sections, we firstly describe the mutation datasets and the features used in the study, and the Random Forest algorithm for constructing the predictive model, PROTS-RF (PROtein Thermostability Random Forest model). We then present the results from cross validation on a single-point mutation dataset and benchmark tests on a set of double-point mutations and a set of multiple point mutations. We test the robustness of the predictive model using hypothetical reverse mutations. We also present a comparison of PROTS-RF to several other relevant potentials or algorithms. In all cases, PROTS-RF delivers better performance than other algorithms. Conclusions and prospects will be presented in the end of the report.

## Materials and Methodology

### Mutation datasets

Three mutation datasets are used in this work. The first dataset was originally collected by Potapov et al. [32]. It contains 2,156 single point mutations (D2156) with experimentally determined changes of folding free energies (ΔΔ*G*). These mutants are derivatives from 84 wild-type proteins. We cluster these proteins using Blastclust [33] with 30% sequence identity and then group these clusters into 5 portions with each having a similar number of mutations. Therefore, proteins from different portions share 30% or less sequence identity. These five groups are then used in a standard five-fold cross validation (CV). The second dataset includes 180 double point mutations (D180) from 27 wild-type proteins with ΔΔ*G* values, was collected by Huang and Gromiha [23]. The final dataset contains 141 multiple point mutations (D141) from 19 different wild type proteins which were collected from ProTherm database [34].

For each mutation in the all three datasets, a corresponding hypothetical reverse mutation (i.e. WT−>MT) is created by swapping the wild-type protein and its mutant involved in the mutation. The free energy change during a hypothetical reverse mutation has the same value but opposite sign to that of the experimental forward mutation (Eq. 1). The hypothetical reverse mutations are grouped in the same fold as their corresponding mutations in the cross validation test. Therefore another benefit of using hypothetical reverse mutations is that the dataset is now perfectly balanced.

### Features

We assemble a set of 41 sequential and structural features. These features are carefully selected so that the free energy can be treated as thermodynamic parameters. The name and description of each feature is available in Table 1. These features can be classified into the following four groups:

**Table 1:** The features and their distributions in the training dataset.

Feature class	Feature	Median		Mean		p-Value (K-S test)	Description
		SM	DM	SM	DM		
Secondary structure & solvent accessibility	Helix	0	0	0.418	0.335	7.5×10^−3^	The secondary structure of wild-type residue.
	Sheet	0	0	0.201	0.308	2.5×10^−4^	
	Coil	0	0	0.381	0.357	0.95	
	Exposed	1	0	0.685	0.478	6.0×10^−15^	The solvent accessibility of wild-type residue.
	Buried	0	1	0.315	0.522	6.0×10^−15^	
Relative difference	POSI	0	0	−0.00528	−0.0327	0.259	Composition difference of positive charged residues (RKH)
	CHAR	0	0	−0.0609	−0.0469	1.3×10^−4^	Composition difference of charged residues (RKHDE)
	SMAL	0	0	−0.113	−0.0427	1.8×10^−4^	Composition difference of small residues (T and D)
	TINY	0	0	0.0661	0.167	2.2×10^−14^	Composition difference of tiny residues (A, G, P, S)
	dASA	0.000350	−0.0159	−0.00153	−0.0179	2.2×10^−16^	Difference of the average of the maximum solvent accessible surface area.
	pIa	0.0023	0.000400	0.00657	0.00114	1.1×10^−8^	Difference of the average pI on all residues.
Evolutionary information	Wtlo	0.0300	0.0400	0.0329	0.0394	2.7×10^−12^	The log-odds of wild-type residue in PSSM
	Wtwt	0.170	0.280	0.306	0.367	1.6×10^−15^	The weighted-score of wild-type residue in PSSM
	Mulo	0	−0.00990	0.000163	−0.0102	2.4×10^−13^	The log-odds of mutant residue in PSSM
	Muwt	0.0100	0.0200	0.0969	0.0562	3.1×10^−11^	The weighted-score of mutant residue in PSSM
	wtlo5	3.80	4.00	3.82	3.74	0.56	The averages of the log-odds of 5 neighboring residues to the WT residue.
	wtwt5	33.7	33.6	35.5	36.6	0.25	The averages of the weighted-score of 5 neighboring residues to WT residue.
	wtlo9	3.90	4.00	3.74	3.84	0.13	The averages of the log-odds of 5 neighboring residues to the WT residue.
	wtwt9	34.8	34.8	35.4	36.7	0.25	The averages of the weighted-score of 5 neighboring residues to WT residue.
	wtlo15	4.07	4.00	3.86	3.79	5.5×10^−3^	The averages of the log-odds of 15 neighboring residues to the WT residue.
	wtwt15	35.4	34.5	36.2	36.1	0.069	The averages of the weighted-score of 15 neighboring residues to WT residue.
PROTS features	FBocc	0.0134	−0.0286	0.012	−0.0271	2.2×10^−22^	The potential difference from the occurrence of continuous tetra-peptide fragments
	FBhel	0.00430	0.00100	0.00292	0.00210	0.085	The potential difference from the occurrence of continuous tetra-peptide fragments which in helix, sheet, coil, buried, exposed or intermediate status.
	FBshe	0.00250	−0.0152	0.00296	0.00147	0.73	
	FBcoi	0.00350	−0.000400	0.00141	−0.00111	0.012	
	FBexp	0.00540	−0.000300	0.00428	−0.00101	5.11×10^−8^	
	FBbur	0.00100	0.00100	0.000984	0.00108	0.95	
	FBint	0.00410	0.00205	0.00336	0.00165	0.042	
	FDhel	0.0320	0.0792	0.0612	0.0917	0.82	The propensity difference of continuous tetra-peptide fragments which in helix, sheet, coil, buried, exposed or intermediate status.
	FDshe	−0.0246	−0.00115	−0.0443	0.00210	0.77	
	FDcoi	0.0550	−0.0243	0.0443	0.00530	0.28	
	FDexp	0.0737	−0.0460	0.0773	−0.0186	2.5×10^−4^	
	FDbur	−0.0788	0.0213	−0.0876	0.0467	0.043	
	FDint	0.0606	0.0590	0.0715	0.0710	0.86	
	FBDTocc*	0.0112	−0.0608	0.0188	−0.0719	9.0×10^−15^	The entropy difference from the occurrence of Delaunay four-residue fragments
	FBDTD43*	0.00975	−0.0953	0.0117	−0.103	2.2×10^−16^	The entropy difference from the occurrence of Delaunay four-residue fragments with at least 3 sequentially continuous residues, only 2 continuous residues and four non-neighboring residues, respectively.
	FBDTD2*	0.00140	−0.0287	0.0134	−0.0365	9.2×10^−13^	
	FBDTD1*	0	0	0.000680	−0.0100	1.6×10^−7^	
	FBDTDD43*	−0.00345	0.00300	−0.00271	0.00271	1.4×10^−3^	The propensity difference of Delaunay four-residue fragments with at least 3 sequentially continuous residues, only 2 continuous residues and four non-neighboring residues, respectively.
	FBDTDD2*	−0.00805	0.00650	0.00736	0.00742	0.16	
	FBDTDD1*	0	0	0.00550	−0.00746	0.067	

The p-values are calculated using the Kolmogorov-Smirnov test (K-S test). Boxplots of these features are available in Figure S1.

* Structure-based features. SM: stabilizing mutations; DM: destabilizing mutations.

#### 1. Evolutionary information (10 features)

PSIBLAST is used to search the wild type proteins against the NCBI non-redundant (NR) protein database pre-filtered by sequence identity of 90% [33]. We consider the log-odds and weighted scores of the wild type residues and mutant residues, as well as the conservation of wild-type residues and neighboring residues in a window centered in the mutation site. We use three different window sizes: 5, 9 or 15. The log-odds and the weighted scores are directly extracted from the position specific scoring matrices (PSSMs) for single point mutations. For multiple point mutations, the averages of these values are used instead. Overall, ten parameters are generated to record the evolutionary information for each single- or multiple- point mutation.

#### 2. Secondary structure and solvent accessibility (5 features)

We assign secondary structure and solvent exposure status of each residue based on the wild-type proteins. If the structure of a wild-type protein is available, we use DSSP [35] to assign the secondary structures of all residues to three states: helix (H), extend (E) and coil (C); and solvent accessibility to exposed (e) or buried (b) using 25% relative accessible surface area as the threshold. We assume that the mutations do not significantly change the conformation of the protein and therefore the secondary structure and the solvent accessibility of wild-type and mutant remain the same.

#### 3. Relative difference (6 features)

We also utilize six relative differences of compositions and properties between the wild-type and the mutant sequences including the change of positive charged residues, charged residues, small residues, tiny residues, maximum area of solvent accessibility (ASA) and the iso-electric point (pIa). These features were identified and used to build a model for discriminating thermophilic proteins from their mesophilic homologs [31].

#### 4. PROTS terms (20 for structure-based model or 13 for sequence-based model)

PROTS is a protein stability potential derived from a comparative study between a large set of thermophilic and mesophilic proteins and a set of point mutations with measurements of mutation induced the change of melting temperature [26]. There are 20 features in this category, including 13 sequential features and 7 Delaunay Tetrahedron (DT) based spatial features if the protein structure is available. The sequential features are used for all models but the Delaunay Tetrahedron based features are only used for structure-based models.

### Random Forest algorithm (RF)

Predictive models are built using the Random Forest algorithm (RF) [36], an ensemble technique utilizing hundreds or thousands of independent decision trees to perform classification or regression. Each of the member trees is built on a bootstrap sample from the training data using a random subset of available variables. The algorithm is a state-of-the-art machine learning method and has been successfully used to build many predictive models [37]–[41]. Unlike many other competitive machine learning algorithms such as support vector machine, RF does not require fine-tuning parameters because using the default values of the parameters often results in near-optimal performance. Moreover, the predicting time for a RF model is often a small fraction of that for a corresponding support vector machine (SVM) model [39]. Another advantage of RF is that it provides several variable importance measures [40], [41]. It is particularly suitable for mining high-dimensional and noisy data. In this study, we use an R implementation of the Random Forest algorithm to construct the predictive model in regression manner [42]. The predicted free energy changes are then used to calculate the accuracy of the predictions using zero change as the threshold for classification.

### Algorithms used for comparison

We compare PROTS-RF to a variety of methods including several top-ranked ones in a recent comprehensive evaluation of protein stability predictors [28]. LSE is a local structure entropy derived from representative protein structures and has shown a strong correlation with protein thermostability [12]. MUpro is a support vector machine (SVM) based predictor at sequence level for the variation of folding free energy (ΔΔ*G*) upon point mutations [18]. I-Mutant2.0 is a SVM based predictor using structure and sequence information for ΔΔ*G* prediction [17]. Both EGAD [13] and FoldX [11] are force fields parameterized on a large set of point mutations with experimentally determined stability changes.

### Evaluation parameters

We use several metrics to measure the performance of the predictive models. The first is accuracy, which is defined as the ratio of the number of correctly predicted mutations in stabilizing or destabilizing of wild type proteins against the total number of predicted mutations. The second is the area under receiver operating characteristic curve (ROC), known as AUC. It should be pointed out that AUC can be a misleading parameter in some situations and therefore the AUC results should be interpreted with caution [43], [44]. We provide AUC for comparison purposes because it is widely used in similar studies. The third is the Pearson correlation coefficient of predicted and experimental ΔΔ*G* values.

## Results

### Statistical analysis of the single mutation dataset

We analyze the statistical distributions of features used in the study. We use the Kolmogorov-Smirnov test for normality find that none but one of the features are normally distributed. We calculate the medium, the mean, and the *p*-value of the Kolmogorov-Smirnov test for each feature's distributions in stabilizing *vs.* destabilizing mutations (Table 1). We also generate boxplots to illustrate the distributions of features of stabilizing and destabilization mutations (Figure S1). The results presented in Table 1 clearly show that the distributions of a number of features are significantly different in stabilizing and destabilizing mutations. For example, mutations occurring in sheets are more likely to be destabilizing (*p*-value: 2.5×10^−4^). Mutations on buried residues are more likely destabilization than stabilization (*p*-value: 6.0×10^−15^), which can be explained by the fact that the protein cores are tightly packed and thus it is difficult to further optimize the interactions within the cores [45].

### Cross validation and model training

**Figure 1:**
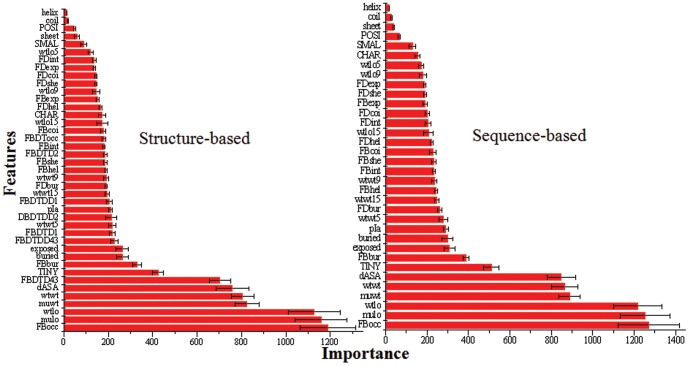
The importance of each feature contributed to the regression predictive models in cross validation. The error bars denote the variation in five-fold cross validation.

We use an R implementation of the Random Forest algorithm to build models. Each model in the five-fold cross validation comprises 2,000 decision trees. The importance of a feature is estimated using the sum of the impurity increase over all trees induced by the feature in the model [36]. The average and standard error of the importance of the 41 features in structure-based prediction and the 34 features in sequence-based prediction are shown in Figure 1. The results clearly show that the PROTS features and the evolutionary information are strongly correlated with protein stability.

The results from all five test datasets in the cross validation are combined. The data from actual experimental and hypothetical mutation are separated and fitted to the experimental data, discretely (Table 2). For the experimental mutations, the Pearson correlation coefficients (R) are 0.628 for the structure-based predictions and 0.620 for the sequence-based predictions (Table 2). We then use various ΔΔ*G* values as cutoff thresholds to classify mutations as stabilizing and destabilizing and calculate the areas under receiver operating characteristic (ROC) curves. We find the areas under ROC (AUC) reach 0.873 and 0.869 for structure and sequence-based predictions, respectively. Very similar R and AUC are obtained for the hypothetical reverse mutations (Table 2). This result demonstrates that the predictive model is quite robust.

**Table 2:** Comparison of prediction performance in cross-validation test.

Methods	WT−>MT			MT−>WT		
	AUC	ACC	R	AUC	ACC	R
MUpro	0.687	0.813	0.483	0.564	0.273	0.167
I-Mutant2.0	0.694	0.775	0.540	0.557	0.683	0.069
LSE	0.577	0.614	0.155	0.577	0.614	0.155
FoldX^a^	0.738	0.714	0.497	-	-	-
EGAD^a^	0.745	0.732	0.595	-	-	-
PROTS (Structure based)	0.819	0.788	0.402	0.819	0.788	0.402
PROTS (Sequence based)	0.815	0.788	0.387	0.815	0.788	0.387
PROTS_RF (Structure based)	**0.873**	**0.799**	**0.628**	**0.863**	**0.795**	**0.622**
PROTS_RF (Sequence based)	**0.869**	**0.794**	**0.620**	**0.858**	**0.796**	**0.616**

a Prediction values were provided by Potapov et al. [32].

AUC: area under ROC curve; ACC: accuracy; R: Pearson Correlation Coefficient.

The model constructed in this work yields comparatively more reliable predictions than other tested models (Table 2). Machine learning based algorithms MUPro and I-mutant2.0 perform poorly for the hypothetical reverse mutations because the AUCs are only slightly higher than 0.5, the level of random selection. The models based on force-fields or potentials such as LSE, FoldX and EGAD can treat temperature and free energy as thermodynamic parameters. The performance of these tested algorithms in the study, nevertheless, are not as good as the PROTS-RF. Besides, PROTS-RF performs better than PROTS, a fragment-based protein thermostability potential we recently developed [26].

We then build the final structure- and sequence- based models using all the 2,156 point mutations and test these models using double- and multiple- point mutations.

### Blind test on double point mutation dataset D180

**Figure 2:**
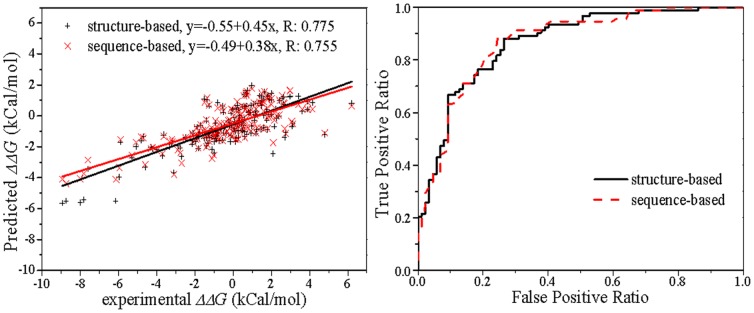
Linear regression and classification of the 180 double point mutations.

In the blind test on the 180 double-point mutations, the regression of prediction against experimentally measured ΔΔ*G* values results in correlation coefficients of 0.775 and 0.755 for structure and sequence-based predictions respectively, and the classification achieves AUCs of 0.868 and 0.869 (Table 3 and Figure 2). The predictions on the experimental data are similar to a previous reported model WET [23], in which the authors achieved correlation coefficients up to 0.75 and the AUC up to 0.87 in 10-fold cross validation tests using a weighted decision table method. However, PROTS-RF achieves very similar results for the hypothetical reverse mutations (0.863 and 0.868 respectively), while the WET model provided by Huang et al. [23] delivers an AUC of 0.518 and R of 0.110, a strong indication for the existence of an over-fitting problem with the model.

**Table 3:** The performance of *ΔΔG* prediction by PROTS-RF for mutations and hypothetical reversed mutations in the D180 dataset, and compare with the WET model.

Dataset		D180	
Mutation directions		WT−>MT	MT−>WT
Structure-based predictions	AUC	0.868	0.863
	ACC	0.782	0.780
	R	0.775	0.774
Sequence-based predictions	AUC	0.869	0.868
	ACC	0.798	0.797
	R	0.755	0.757
WET	AUC	0.961	0.518
	ACC	0.85	0.572
	R	0.930	0.110

AUC: area under ROC curve; ACC: accuracy; R: Pearson Correlation Coefficient.

Huang et al suggested that the methods developed for predicting protein stability change upon single point mutations may not be suitable for predicting the stability change upon double point mutations because the thermostability changes are not always additive [23]. Our results, nevertheless, have clearly indicated that a predictive model trained from single point mutations may still be capable of predicting double point mutations induced by protein stability changes. Some features used in our models, especially PROTS terms, reflect the surrounding environment of the mutation sites. The changes of these features are additive for remote mutations but not additive for mutations close to each other. This approach is consistent with the observations that in general non-additive mutations involve mutations close to each other while additive mutations involve mutations far apart (There are exceptions, however, to this rule because of long range interactions).

### Blind test on multiple point mutations D141

**Figure 3:**
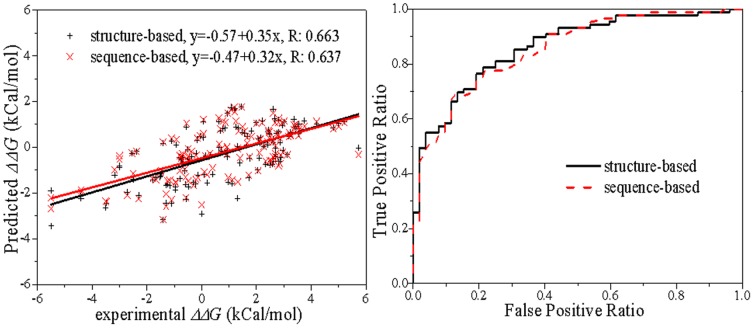
Linear regression and classification of the 141 multiple point mutations.

The thermostability changes upon multiple point mutations are more complicated than single- and double- point mutations and therefore it is expected to be more difficult to be correctly predicted. Nevertheless, the correlation coefficients of predictions of the 141 multiple point mutations and experimentally measured ΔΔ*G* values reach 0.663 and 0.637 for structure and sequence-based predictions, and the classification results in AUCs of 0.862 and 0.855, respectively (Figure 3 and Table 4). This result suggests that our predictive model is also capable of predicting stability changes upon multiple point mutations with high accuracy.

**Table 4:** The performance of *ΔΔG* prediction by PROTS-RF for mutations and hypothetical reversed mutations in the D141 dataset.

Dataset		D141	
Mutation directions		WT−>MT	MT−>WT
Structure-based predictions	AUC	0.862	0.858
	ACC	0.787	0.789
	R	0.663	0.659
Sequence-based predictions	AUC	0.855	0.844
	ACC	0.779	0.746
	R	0.637	0.629

AUC: area under ROC curve; ACC: accuracy; R: Pearson Correlation Coefficient.

### Prediction thermostability of Staphylococcal Nuclease mutants

**Figure 4:**
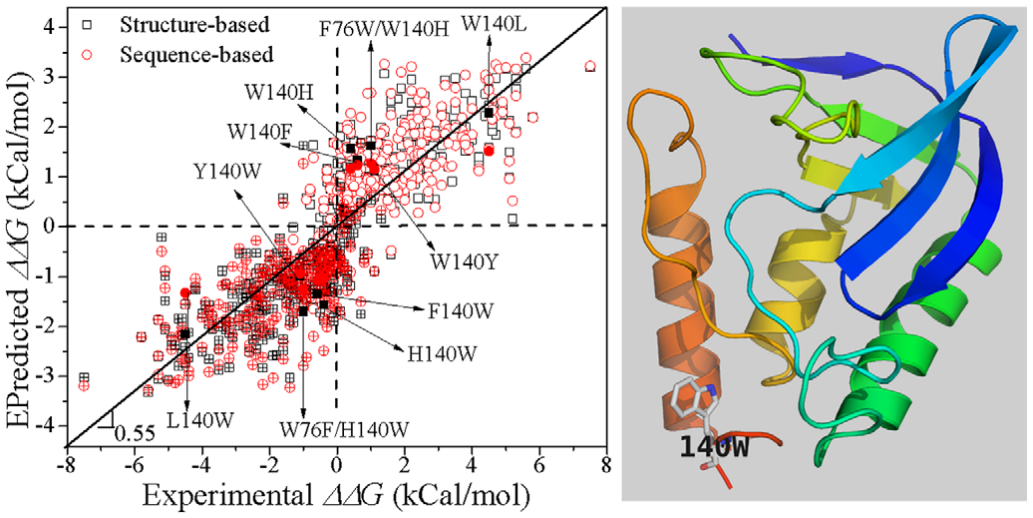
Structure and sequence based prediction of mutations of staphylococcal nuclease. Empty symbols are prediction for mutations with experimental data, and the corresponding crossed-symbols are the prediction for hypothetical reverse mutations. The structural figure is based on the PDB entry 1STN.

Staphylococcal Nuclease (SNase) has been used as a model protein for studying protein stability and therefore there is a significant amount of experimental data for free energy changes upon mutations of this enzyme [46]. We use PROTS-RF predict free energy changes upon mutations and then plot them against the experimental values in Fig. 4. The predicted and experimental ΔΔ*G* values narrowly distribute along a line passing through the Origin. Both structure-based and sequence-based predictions are highly correlated with the experimental data (R_Pearson_ = 0.855 and 0.843, respectively), and the predictions for mutations and the corresponding hypothetical reverse mutations are strongly symmetric with respect to the Origin. A Trp residue at position 140 is critical to SNase structure, stability and function [47]. PROTS-RF correctly predicts W140 related mutations and their hypothetical reverse mutations qualitatively but not quantitatively (Fig. 4), suggesting further improvement remains desirable.

## Discussion

The model developed in the study is robust as demonstrated in the cross validation and blind tests. We believe that the high robustness of this model can be attributed to the Random Forest algorithm and the features used in the models. The Random Forest algorithm is well known for its high robustness and is particularly suitable for mining high-dimensional and noisy data. We utilize diverse features ranging from evolutionary information, protein structure profile, and protein properties to the thermostability terms learned from a large amount of native proteins [26], [31]. These features are less dependent on the proteins in training datasets and the over-fitting problem is less pronounced in the model. Consequently, they are robust and capable of predicting not only single-point mutations, but also double- or multiple- point mutations. The tests using the hypothetical reverse mutations in this study have shown that the tested machine learning models for predicting mutation induced protein stability change may suffer from the over-fitting problem. The results are surprising because all these models have undergone cross validation, a common practice widely considered as a rigorous validation approach. We suggest that testing datasets created based on physical principles can be highly useful for testing the robustness of predictive models.

In the present study, it is observed that the structure-based and sequence-based predictors result in very similar performance, suggesting the structural features used in the study do not make significant contribution to the performance of the models. This is consistent to their relatively low importance as shown in Figure 1. The most important structural feature (FBDTD43) is the seventh overall most important feature. Its ability to deliver good predictions without structural information is advantageous over other methods requiring structural information because vast majority of proteins do not have solved structures. It is possible that the information encoded in these structural features is also captured in the sequential features used in the study. In addition, the number of structural features is relatively small (7 structural vs. 34 sequential features) and they may not interact well with sequential features. Nevertheless, we think it is possible to further improve model performance if the structural class of proteins and more structure-based features are considered. Recently, we were made aware that alpha/beta class proteins normally have higher residue contact density (i.e., number of contacts per residue) than other proteins [48]. Proteins with higher contact density tend to bear more mutations without significantly change its thermostability [49] and thermophiles tend to have higher contact density than mesophiles [50]. Moreover, a recently report concluded that the accessible surface area of beta proteins increases more rapidly with the size of proteins in comparison with that of the alpha proteins [51]. It was also reported that the aggregation propensity of a protein is highly correlated with its structural classification [52]. Currently we are investigating different classes of proteins and will report the results in future.

## Conclusion

We have presented PROTS-RF, a predictive model based on the Random Forest algorithm for predicting mutation induced protein stability change. This model is constructed based on a large set of features in proteins and trained by the Random Forest algorithm. In the cross validation test and the blind tests using double- and multiple- mutation datasets, this model is comparatively more reliable in the prediction of protein thermostability changes over other existing methods. It also shows high levels of robustness in the tests using hypothetical reverse mutations. We demonstrate that the hypothetical reverse mutations based on physical principles are highly useful for testing the robustness of algorithms for predicting mutation induced protein stability change.

## Supporting Information

Figure S1 **The distributions of features of stabilizing and destabilization mutations.**
(TIF)
