# Supplementary figures and images for: PROTS-RF: A Robust Model for Predicting Mutation-Induced Protein Stability Changes
Source: PLoS One. 2012 Oct 15;7(10):e47247. doi: 10.1371/journal.pone.0047247 (PMC3471942; doi:10.1371/journal.pone.0047247)

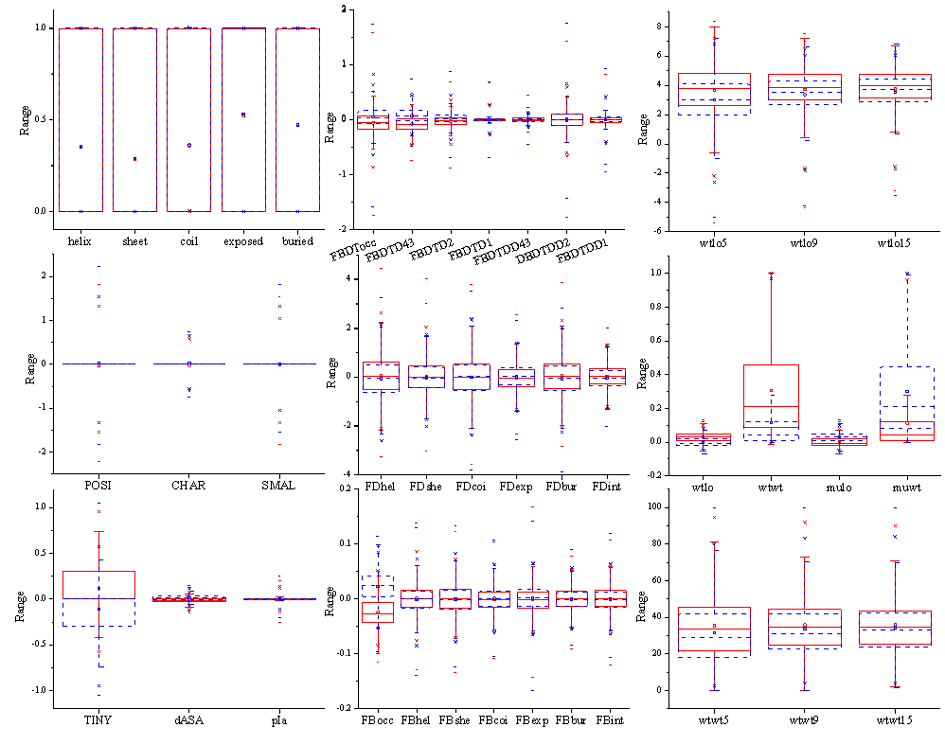

Supplement: Figure S1 — The distributions of features of stabilizing and destabilization mutations. (TIF) [file pone.0047247.s001.tif]
